# Carbon variation of dry grasslands in Central Asia in response to climate controls and grazing appropriation

**DOI:** 10.1007/s11356-022-18542-2

**Published:** 2022-01-11

**Authors:** Shihua Zhu, Xi Chen, Chi Zhang, Xia Fang, Liangzhong Cao

**Affiliations:** 1 Jiangsu Climate Center, Nanjing, 210009 China; 2 International Institute for Earth System Science, Nanjing University, Nanjing, 210093 China; 3 State Key Laboratory of Desert and Oasis Ecology, Xinjiang Institute of Ecology and Geography, Chinese Academy of Sciences, Urumqi, 830011 China; 4 Shandong Provincial Key Laboratory of Water and Soil Conservation and Environmental Protection, College of Resources and Environment, Linyi University, Linyi, 276000 China; 5 Research Center for Ecology and Environment of Central Asia, Chinese Academy of Sciences, Urumqi, 830011 China; 6 Xinjiang Institute of Engineering, Urumqi, 830091 Xinjiang China; 7 Jiujiang University, Jiujiang, 332005 China

**Keywords:** Carbon dynamics, AEM grazing model, Grazing, Climate change, Central Asia, Precipitation

## Abstract

Quantification of grassland carbon (C) variations is necessary for understanding how grazing and climate change interact to regulate carbon capture and release. Central Asia (CA) has the largest temperate grassland belt in the world and unique temperate dryland ecosystems, which experienced severe climate change and grazing-induced disturbances. However, the impact of grazing on C dynamics is highly uncertain owing to climate variations. Here, an arid ecosystem model (AEM) supplemented with a grazing module that specifically addressed physiological and ecological characteristics of dryland vegetation was developed to quantitatively simulate grassland C dynamics in response to changes in precipitation, temperature, grazing intensity, and CO_2_ level in the past decades. The regional simulation results showed that net primary productivity (NPP) was affected mainly by precipitation (in 59% of the studied area). Grazing had a negative effect on NPP and C stocks, whereas overcompensation occurred in 25.71% of the studied area, mainly in the dry western parts. The complex interaction effects of climate, CO_2_, and grazing negatively affected productivity, with a grassland NPP decrease of − 1.14 g C/m^2^/a and high interannual variability. We found that the temporal pattern of cumulative C sequestration, especially total C and vegetation C (VEGC), closely followed the annual fluctuations of precipitation. VEGC stocks decreased from 182.22 to 177.82 g C/m^2^, with a very low value between 1998 and 2008, when precipitation significantly decreased. The results indicate that southern Xinjiang and the Turgay Plateau of Kazakhstan are ecologically fragile areas due to grassland degradation.

## Introduction

Grassland ecosystems are the most widespread types of vegetation worldwide, accounting for approximately 40% of terrestrial land areas and yielding 35% of global plant growth (Zhou et al. 2017; Zhang et al. 2019). Previous studies have indicated that grasslands have a strong carbon sequestration potential, but they are disturbed by climate change and grazing (Qiu et al. 2013; Zhu et al. 2016). Temperate grasslands play an indispensable and increasingly predominant role in the global carbon (C) cycle (Scurlock and Hall 1998; Han et al. 2016). Although the impact of grazing on grassland C balance and dynamics is still uncertain, it is generally believed that overgrazing is harmful to vegetation communities, and that this negative effect reduces the potential grassland productivity by a third. Consequently, the huge surface area of grasslands (nearly 9 × 10^6^ km^2^ in temperate zones) and their dominant role in the trends and interannual variations of global terrestrial C dynamics make them critical in studies of the effect of global climate changes on C budget (Ahlström et al. 2015; Zhu et al. 2020).

Central Asia (CA) is located in the hinterland of Eurasia and has unique temperate dryland ecosystems and the largest temperate grassland belt in the world (Chen et al. 2017a). Temperate grasslands in CA are regions with a fragile environment, intensive human activity, limited resources, and relatively scarce ecological services (Zhu et al. 2020). Compared to C dynamics in other terrestrial ecosystems, the C cycle CA grasslands is more sensitive to precipitation due to moisture restrictions (Lioubimtseva and Henebry 2009; Huang et al. 2016). Rainfall or soil water content is usually the most important factor limiting photosynthesis and respiration in grassland ecosystems (Sala et al. 1988), especially in arid and semi-arid ecosystems (MacNeil et al. 2008). Grazing directly and indirectly affects ecological processes through the redistribution of biomass and nutrients (Frank et al. 2002). C dynamics of grassland ecosystems is mainly affected by grazing in the following ways: (1) change in the efficiency of light, (2) reduction of water loss and water stress, (3) acceleration or changes in the nutrient cycle, (4) redistribution of biomass, and (5) changes in the photosynthesis rate (Leriche et al. 2001; Chen et al. 2007; Han et al. 2016). The positive or negative effects of grazing on vegetation often uniquely depend on the climate factors. Temperate dryland grasslands are co-regulated by temperature, precipitation, CO_2_ level, and man-induced disturbances (e.g., grazing), with different ecosystems being affected by distinct factors (Zhu et al. 2020). The C source/sink characteristics may reverse under the pressure of dramatic environmental disturbance (Ciais et al. 2005) or improper human utilization of natural resources, such as overgrazing (Han et al. 2016), deforestation, and urbanization (Costanza et al. 2014), which makes the regional C budget greatly uncertain.

In recent decades, the grassland desertification in CA has increased at an annual rate of 0.1–0.7% (Parey et al. 2007). Overgrazing contributes a lot to grassland degradation or desertification, and climate variability accelerates this process. The combined effects of grazing and climate factors may produce positive or negative C-atmosphere feedback, which may lead to either the amplification or attenuation of the grazing effect (Zhou et al. 2017). However, the exact impact of grazing on grassland productivity is still unclear, with some studies demonstrating stimulatory (Klein et al. 2007), suppressive (Wu et al. 2008), or no significant effects (Biondini et al. 1998). Hence, describing the magnitude and pattern of C dynamics in CA under the dual pressure caused by climate change and grazing, quantitatively identifying and isolating the individual and interaction effects of different factors (including climate, CO_2_ level, and grazing) on C variation, and setting a grazing intensity threshold should form the basis for regional sustainable development and ecological measures to improve the state of the environment (McSherry and Ritchie 2013).

At present, data on the individual and combined impacts of grazing and climate change on C variations in CA grassland ecosystem are urgently needed. We searched research papers published in 1980–2021 and found that few studies focused on arid and semi-arid grasslands in CA. The few field (Alimaevi et al. 2008) or model studies (Han et al. 2016; Chen et al. 2017b, 2018) lacked a description of the special physiological and ecological characteristics of the vegetation in arid areas (high root-to-shoot ratio, vertical root distribution, etc.). Therefore, in the present study, we have developed an arid ecosystem model (AEM) with a grazing module. The model has been significantly optimized in terms of dry vegetation structure, water and salt transportation methods, and other factors. Moreover, our AEM has been applied to characterize C dynamics of different plant functional types in dryland (Zhang et al. 2013; Li et al. 2015; Fang et al. 2017; Zhu et al. 2019). The present study was performed to (1) assess the temporal and spatial patterns of grassland C dynamics in CA and its response to multiple environmental factors; (2) quantify individual and combined effects of grazing and climate variation on grassland C dynamics as well as the interaction between climate, CO_2_ level, and grazing; and (3) identify ecologically vulnerable areas in CA.

## Materials and methods

### Study region

**Fig. 1:**
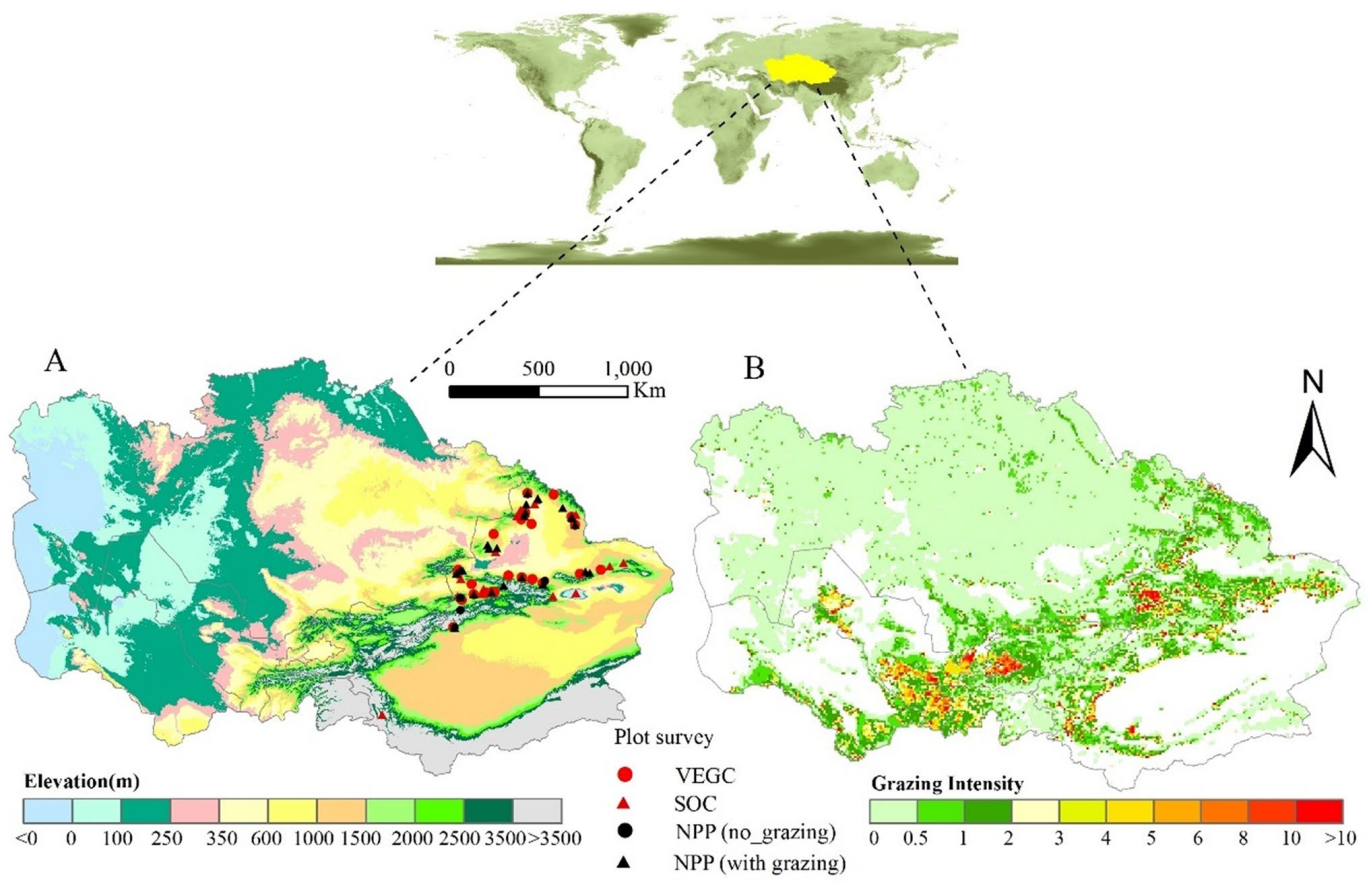
Study area. (A) Elevation and the characteristic land features in CA. (B) The grazing intensity pattern in CA in 2005 (Food and Agriculture Organization). The red and black cycles and triangles denote sampling plots with VEGC, SOC, NPP (no_grazing), and NPP (with grazing)

CA (34.3–55.4° N, 46.5–96.4° E) lies deep in the hinterland of Eurasia and consists of the Xinjiang Province and five Asian states (Kazakhstan, Kyrgyzstan, Tajikistan, Turkmenistan, and Uzbekistan) (Hu et al. 2014). The unique mountain-oasis-desert ecosystem pattern of this region is of global significance. Approximately 50% of CA is covered by grasslands, including alpine meadows, steppes, and desert grasslands (Liu et al. 2016). The pastures extend from the edge of deserts at 400 m above sea level to alpine meadows at 3500 m in the high mountainous areas (Fig. 1). Following the increasing elevation gradient, the annual mean temperature varies from 15 to − 3 °C, whereas the mean annual precipitation varies from 140 to 600 mm. Typical plant species found in the mountain meadows are *Bromus inermis*, *Poa pretensis*, and *Roegneria kamoji Ohwi*; typical steppe plants are *Festuca ovina L.*, *Stipa capillata*, and *Stipa glareosa P. A. Smirn*; and typical steppe desert plants are *Seriphidiam santolinum (Schrenk) Poljak*, *Sympegma regelii*, and *Reaumuria soongonica (Pall.) Maxim*.

### Model description

#### AEM

Our AEM couples biogeochemical and biophysical processes and has been shown to perform well in modeling dryland ecosystems (Zhang et al. 2013). The advantage of the AEM lies in its ability to accurately emphasize and quantify the structure and special physiological characteristics of desert vegetation. However, the initial AEM model ignored the impact of grazing on the grassland ecosystem. This study included a defoliation equation from Seligman et al. (1992) and a revision of the grazing process after Luo et al. (2012) to develop a grazing module that would make the AEM more applicable to arid grasslands. The details of the background behind the AEM are outlined in Zhang et al. (2013).

#### Grazing module

**Fig. 2:**
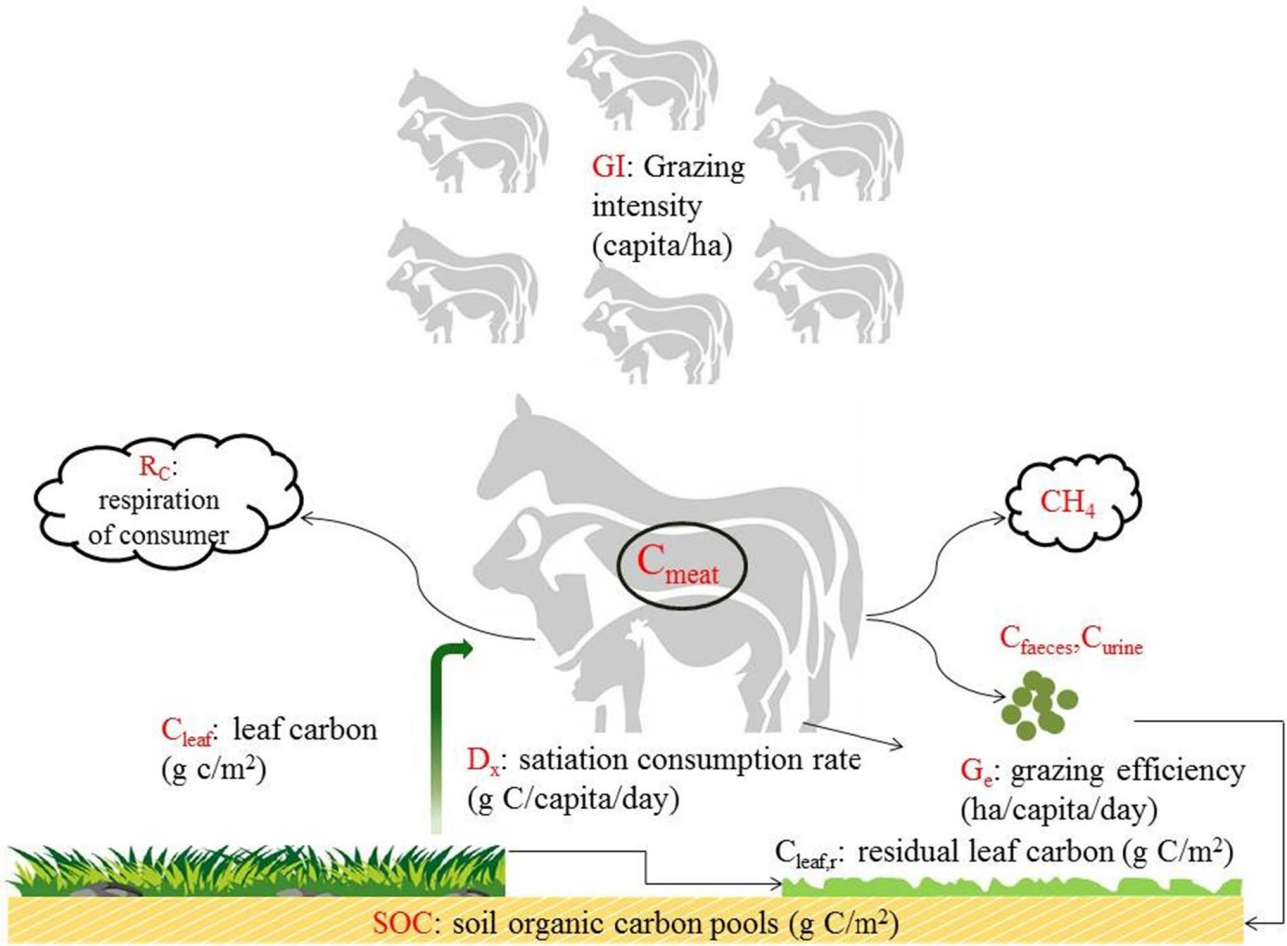
Illustration of the grazing module. Detailed descriptions of the model parameters can be found in Table 1

The grazing module of the AEM incorporated a revised defoliation equation (Luo et al. 2012), which simulated the impact of grazing on grassland C cycle on a daily basis (Fig. 2).

1. **C balance**
  Grazing directly reduces leaf C (C_leaf_) and increases soil organic C (SOC) through feces and urine inputs
  1 $$\Delta {\mathrm{C}}_{\mathrm{leaf}}=-{\mathrm{C}}_{\mathrm{graze}}$$
  2 $$\Delta \mathrm{SOC}={\mathrm{C}}_{\mathrm{faeces}}+{\mathrm{C}}_{\mathrm{urine}}={f}_{\mathrm{faeces}}\times {\mathrm{C}}_{\mathrm{graze}}+{f}_{\mathrm{urine}}\times {\mathrm{C}}_{\mathrm{graze}}$$
  where ${f}_{\mathrm{faeces}}$ and ${f}_{\mathrm{urine}}$ are parameters that determine the fractions of consumed grass C (${\mathrm{C}}_{\mathrm{graze}}$) that are converted to feces and urine. The consumed C that does not return to the ecosystem as ${\mathrm{C}}_{\mathrm{faeces}}$ and ${\mathrm{C}}_{\mathrm{urine}}$ is lost as CO_2_, CH_4_, and meat/dairy products:
  3 $${\mathrm{C}}_{\mathrm{loss},\mathrm{graze}}={R}_{c}+{\mathrm{CH}}_{4}+{\mathrm{C}}_{\mathrm{meat}}={f}_{Rc}\times {\mathrm{C}}_{\mathrm{graze}}+{f}_{\mathrm{CH}4}\times {\mathrm{C}}_{\mathrm{graze}}+{f}_{\mathrm{meat}}\times {\mathrm{C}}_{\mathrm{graze}}$$
  where ${\mathrm{C}}_{\mathrm{loss},\mathrm{graze}}$ is the total C loss, ${R}_{c}={f}_{Rc}\times {\mathrm{C}}_{\mathrm{graze}}$ is the C lost through consumer (i.e., livestock) respiration,${\mathrm{CH}}_{4}={f}_{\mathrm{CH}4}\times {\mathrm{C}}_{\mathrm{graze}}$ is the C released by livestock in the form of methane, and ${\mathrm{C}}_{\mathrm{meat}}={f}_{\mathrm{meat}}\times {\mathrm{C}}_{\mathrm{graze}}$ is the C exported as meat/dairy products. The residential time of the meat/dairy product pool is 1 year.
  The parameter values of ${f}_{\mathrm{faeces}}$, ${f}_{Rc}$, and ${f}_{\mathrm{CH}4}$ were determined by several former studies (Schimel et al. 1986; Minonzio et al. 1998) (see Table 1). The value of ${f}_{\mathrm{urine}}$ was estimated as follows:
  4 $${f}_{\mathrm{urine}}=\frac{{C}_{\mathrm{urine}}}{{C}_{\mathrm{graze}}}=\frac{{CN}_{\mathrm{urine}}\times {N}_{\mathrm{urine}}}{{C}_{\mathrm{graze}}}$$
  where ${\mathrm{CN}}_{\mathrm{urine}}=12/28$, i.e., it is the C:N ratio of urea, ${\mathrm{N}}_{\mathrm{urine}}$ is nitrogen (N) in urine. According to Parton et al. (1987), a large proportion (${f}_{\mathrm{N},\mathrm{excreta}}\approx 80\%$) of N (${\mathrm{N}}_{\mathrm{graze}}$) becomes re-sealed and stored in the soil as livestock excrement (${\mathrm{N}}_{\mathrm{excreta}}$). Menzi et al. (1997) found that ${\mathrm{N}}_{\mathrm{urine}}$ accounts for more than half (${f}_{\mathrm{excreta}\_\mathrm{N},\mathrm{urine}}\approx 60\%$) of ${\mathrm{N}}_{\mathrm{excreta}}$. Therefore
  5 $${\mathrm{N}}_{\mathrm{urine}}={f}_{\mathrm{excreta}\_\mathrm{N},\mathrm{urine}}\times {\mathrm{N}}_{\mathrm{excreta}}={f}_{\mathrm{excreta}\_\mathrm{N},\mathrm{urine}}\times {{f}_{\mathrm{N},\mathrm{excreta}}\times \mathrm{N}}_{\mathrm{graze}}$$
  Substituting ${N}_{urine}$ in Eq. (4) with Eq. (5), we obtain
  6 $$\begin{array}{c}{f}_{\mathrm{urine}}=\frac{{\mathrm{CN}}_{\mathrm{urine}}\times {f}_{{\mathrm{excreta}}_{\mathrm{N}},\mathrm{urine}}\times {{f}_{\mathrm{N},\mathrm{excreta}}\times N}_{\mathrm{graze}}}{{C}_{\mathrm{graze}}}\\ =\frac{{\mathrm{CN}}_{\mathrm{urine}}\times {f}_{{\mathrm{excreta}}_{\mathrm{N}},\mathrm{urine}}\times {f}_{\mathrm{N},\mathrm{excreta}}}{{(\mathrm{C}}_{\mathrm{graze}}/{\mathrm{N}}_{\mathrm{graze}})}=\frac{{\mathrm{CN}}_{\mathrm{urine}}\times {f}_{\mathrm{excreta}\_\mathrm{N},\mathrm{urine}}\times {f}_{\mathrm{N},\mathrm{excreta}}}{{\mathrm{CN}}_{\mathrm{leaf}}}\end{array}$$
  where ${\mathrm{CN}}_{\mathrm{leaf}}$ is the leaf C/N ratio. Then, the parameter ${f}_{\mathrm{meat}}$ is estimated as
  7 $${f}_{\mathrm{meat}}=100\mathrm{\%}-{f}_{\mathrm{Rc}}-{f}_{\mathrm{CH}4}-{f}_{\mathrm{faeces}}-{f}_{\mathrm{urine}}$$
2. **Estimation of the actual grass consumption rate**
  The actual ${\mathrm{C}}_{\mathrm{graze}}$ was determined by the balance between the herd’s demand (${\mathrm{C}}_{\mathrm{demand}}$) and forage supply (${\mathrm{C}}_{\mathrm{supply}}$).
  8 $${\mathrm{C}}_{\mathrm{graze}}=\mathrm{min}({\mathrm{C}}_{\mathrm{demand}},{\mathrm{C}}_{\mathrm{supply}})$$
  The daily ${\mathrm{C}}_{\mathrm{demand}}$ was determined by the herd density or grazing intensity (${G}_{I}$) and the sheep satiation consumption rate (${D}_{X}$).
  9 $${\mathrm{C}}_{\mathrm{demand}}={G}_{I}\times {D}_{X}$$
  The satiation consumption rate was defined as the maximum grass consumption rate per capita. According to the National Research Council of the US (NRC 1985), the sheep ${D}_{X}$ was 2,400 g C day^−1^ sheep^−1^. The herd densities of all other livestock types were converted to the sheep-equivalent ${G}_{I}$ based on their specific ${D}_{X}$ values (NRC 1985).

**Table 1:** Model parameters

Parameter	Unit	Value	Description	Source
D_X_	g/day /sheep	2,400	Sheep satiation consumption rate	(NRC 1985)
G_e_	ha/day/sheep	0.011	Sheep grazing efficiency	(Seligman et al. 1992)
C_leaf,r_	g/m^2^	6.75	Residual leaf carbon unavailable to animals	(Seligman et al. 1992)
CN_leaf_	DIM	24	Leaf C:N ratio	(Dong and Yu 2008)
CN_urine_	DIM	0.43	C:N ratio of urine, assuming all urine is in form of urea	(Riedo et al. 2000)
*f*_Rc_	DIM	0.5	Fraction of consumed carbon respired	(Minonzio et al. 1998)
*f*_CH4_	DIM	0.03	Fraction of consumed carbon in CH_4_	(Minonzio et al. 1998)
*f*_faeces_	DIM	0.3	Fraction of consumed carbon in faeces	(Schimel et al. 1986)
*f*_N,excreta_	DIM	0.8	Fraction of consumed nitrogen in excreta	(Parton et al. 1987)
*f*_excreta_N,urine_	DIM	0.6	Fraction of excreted nitrogen in urine	(Menzi et al. 1997)
*f*_urine_^*^	DIM	0.008	Fraction of consumed carbon in urine	(Riedo et al. 2000)
*f*_meat_	DIM	0.16	Fraction of consumed carbon in meat	= 1 − *f*_Rc_ − *f*_CH4_ − *f*_faeces_ − *f*_urine_

^*^ f_urine_ = (1/CN_leaf_) × *f*_N,excreta_ × *f*_excreta_N,urine_ × CN_urine_

The daily ${\mathrm{C}}_{\mathrm{supply}}$ is a function of the daily grazed grassland area (${\mathrm{Area}}_{\mathrm{graze}}$) and grass available to livestock (${\mathrm{C}}_{\mathrm{leaf},\mathrm{av}}$).

10a $${\mathrm{C}}_{\mathrm{supply}}={\mathrm{Area}}_{\mathrm{graze}}\times {\mathrm{C}}_{\mathrm{leaf},\mathrm{av}}$$

10b $${\mathrm{Area}}_{\mathrm{graze}}={G}_{e}\times {G}_{I}$$

10c $${\mathrm{C}}_{\mathrm{leaf},\mathrm{av}}={\mathrm{C}}_{\mathrm{leaf}}-{\mathrm{C}}_{\mathrm{leaf},\mathrm{r}}$$

where ${G}_{e}$ is the mean land area that can be covered by a sheep each day (Seligman et al. 1992), ${\mathrm{C}}_{\mathrm{leaf}}$ is the total leaf C of the grassland, and ${\mathrm{C}}_{\mathrm{leaf},\mathrm{r}}$ is the leaf remains that cannot be consumed by sheep (Seligman et al. 1992).

### Field experiments and model validation

**Fig. 3:**
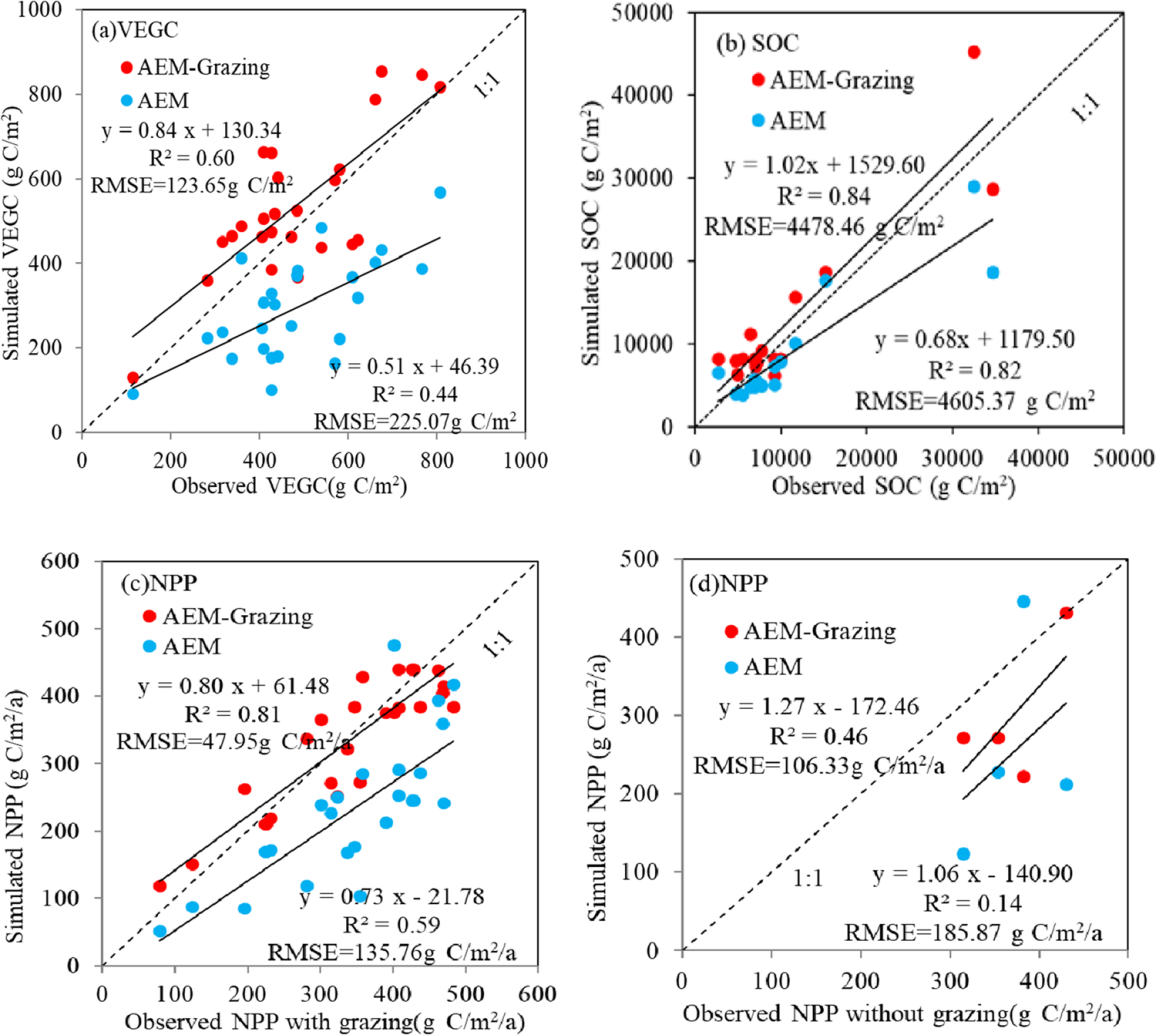
Comparison of C dynamics simulated by the initial AEM and AEM with the grazing module with actual measured values

In previous studies, we conducted AEM sensitivity analyses (Zhang et al. 2013) and quantitatively assessed the responsiveness of the model to environmental factors (Zhang and Ren 2017; Zhu et al. 2019). In this study, we confirmed that the simulation performance of the AEM with the grazing module was better than that of the initial AEM (Fig. 3). To further assess the consistency of C dynamics in CA grassland ecosystems with the obtained results (taking grazing into account), we compared the site simulation results with the observed SOC (17 plots), vegetation C (VEGC; 25 plots), and net primary productivity (NPP; 30 plots, including 26 grazing and four no-grazing scenarios) from a previous survey in Xinjiang and CA (Li et al. 2013, 2015). These validation sites covered representative grassland types in dryland under different grazing conditions (Fig. 1).

### Model inputs and scenario design

#### Meteorological dataset

The climate data required for the operation of the AEM with the grazing module mainly included spatial explicit daily climate datasets (precipitation, relative humidity, solar radiation, and daily minimum, maximum, and mean temperature). Due to the scarcity and uneven distribution of meteorological stations in CA (most are distributed in oases, whereas stations in the vast desert areas are very rare), there will be greater uncertainty in interpolation using the observation data of limited and unevenly distributed meteorological stations (Hu et al. 2014). Climate Forecast System Reanalysis (CFSR) dataset provided by the US National Centers for Environmental Prediction (http://rda.ucar.edu/pub/cfsr.html) was used as it has been shown to have high reliability in the study area (Li et al. 2015). This dataset also has been widely used in climate change research (Saha et al. 2010; Zhang and Ren 2017) and fully verified in CA at site and regional scales (Zhu et al. 2019, 2020). Although that dataset overestimates precipitation in the forested area of the Tianshan Mountains to a certain degree, given that this study was mainly concerned with grassland C dynamics in arid areas, the overestimation of precipitation in forested areas had little impact on AEM performance.

#### Grazing data

The spatial data of grazing intensity were obtained from the Gridded Livestock of the World Project of the Food and Agriculture Organization of the United Nations. The main types of livestock involved in GLW data are cattle, buffalo, sheep, and goats. This study uses the concept of “equivalent sheep” or “standard sheep” to convert the livestock involved. The conversion standard refers to the “People’s Republic of China Agricultural Industry Standard-Calculation of the Reasonable Stocking Capacity of Natural Grassland” (Han et al. 2016):

- 1 cow = 6.0 sheep
- 1 buffalo = 6.5 sheep
- 1 goat = 0.9 sheep

Due to the difficulty of data acquisition, the Food and Agriculture Organization currently provides grazing intensity data only for 2005, 2010, and 2015. Long-time series grazing data from 1980 to 2014 were interpolated and converted according to the number of livestock in CA countries (http://www.fao.org/faostat/en/#home) and Xinjiang, China (http://www.xjtj.gov.cn/sjcx/tjnj_3415/).

#### Scenario design

Simulation of C dynamics in CA consisted mainly of three parts: equilibrium, spin-up, and transient states. First, we set a baseline for C dynamics by running the equilibrium state with the initial driving datasets. Because the reanalyzed meteorological data before 1979 were not available, the average climate data for the pre-study period (1980–1989) were used as equilibrium data (Li et al. 2015; Zhang and Ren 2017; Zhu et al. 2020). In the generation of equilibrium climate, trends of climate variables were removed, while their day-to-day variations were kept. In the spin-up phase, we used long-term detrended meteorological data to drive the model. This was done to reduce the fluctuations in the transition from the equilibrium state to the transient simulation. Finally, six scenarios were created to isolate the effects of individual factors on C dynamics in CA (Table 2).

**Table 2:** Design of the simulation experiments

Experiment	CO_2_	Grazing intensity	Climate		Description
			Precipitation	Temperature^b^	
OVERALL	1980–2014	1980–2014	1980–2014	1980–2014	Overall in reality
No grazing	1980–2014	No grazing	1980–2014	1980–2014	No grazing
CO_2_	1980–2014	1980	Equilibrium^a^	Equilibrium	CO_2_ change
PREC	1980	1980	1980–2014	Equilibrium	Precipitation change
TEMP	1980	1980	Equilibrium	1980–2014	Temperature change
CLIM	1980	1980	1980–2014	1980–2014	Climate change

^a^Equilibrium climate was generated using the average climate data from 1980 to 1989 (Zhang et al. 2013). ^b^Temperature refers to daily maximum, minimum, and average temperatures

In OVERALL scenario, the AEM with a grazing module was driven by historical changes in meteorological parameters, CO_2_ levels, and grazing intensity. This scenario was used to analyze the actual effect of various factors on the grassland C dynamics. In no grazing scenario, historical climate and CO_2_ data were used but grazing was removed during the experiment to describe a “no grazing” scenario. CO_2_, PREC, and TEMP were created to assess the effects of individual factors (CO_2_, precipitation, and temperature) on C dynamics. During the simulation of the individual factor effects, only the analyzed factors were permitted to vary over time, while other factors remained unchanged. In CLIM scenario, the climate changed over time, while CO_2_ level and grazing were kept unchanged (Table 2). The dynamic changes in NPP, total carbon (TOTC), VEGC, and SOC from 1980 to 2014 were calculated by comparing the mean values from 1997 to 2014 and from 1980 to 1997. Based on these data, we carried out factor analyses to explore the individual effects of environmental elements and their dynamic interaction on NPP, TOTC, VEGC, and SOC as follows:

$${\mathbf{O}\mathbf{V}\mathbf{E}\mathbf{R}\mathbf{A}\mathbf{L}\mathbf{L}}_{\mathbf{e}\mathbf{f}\mathbf{f}\mathbf{e}\mathbf{c}\mathbf{t}}={\mathbf{V}\mathbf{A}\mathbf{R}}_{1997-2014\_\mathbf{O}\mathbf{V}\mathbf{E}\mathbf{R}\mathbf{A}\mathbf{L}\mathbf{L}}-{\mathbf{V}\mathbf{A}\mathbf{R}}_{1980-1997\_\mathbf{O}\mathbf{V}\mathbf{E}\mathbf{R}\mathbf{A}\mathbf{L}\mathbf{L}}$$

$${\mathbf{T}\mathbf{E}\mathbf{M}\mathbf{P}}_{\mathbf{e}\mathbf{f}\mathbf{f}\mathbf{e}\mathbf{c}\mathbf{t}}={\mathbf{V}\mathbf{A}\mathbf{R}}_{1997-2014\_\mathbf{T}\mathbf{E}\mathbf{M}\mathbf{P}}-{\mathbf{V}\mathbf{A}\mathbf{R}}_{1980-1997\_\mathbf{T}\mathbf{E}\mathbf{M}\mathbf{P}}$$

$${\mathbf{P}\mathbf{R}\mathbf{E}\mathbf{C}}_{\mathbf{e}\mathbf{f}\mathbf{f}\mathbf{e}\mathbf{c}\mathbf{t}}={\mathbf{V}\mathbf{A}\mathbf{R}}_{1997-2014\_\mathbf{P}\mathbf{R}\mathbf{E}\mathbf{C}}-{\mathbf{V}\mathbf{A}\mathbf{R}}_{1980-1997\_\mathbf{P}\mathbf{R}\mathbf{E}\mathbf{C}}$$

$${\mathbf{C}\mathbf{O}}_{2\mathbf{e}\mathbf{f}\mathbf{f}\mathbf{e}\mathbf{c}\mathbf{t}}={\mathbf{V}\mathbf{A}\mathbf{R}}_{1997-2014\_\mathbf{C}\mathbf{O}2}-{\mathbf{V}\mathbf{A}\mathbf{R}}_{1980-1997\_\mathbf{C}\mathbf{O}2}$$

$${\mathbf{C}\mathbf{L}\mathbf{I}\mathbf{M}}_{\mathbf{e}\mathbf{f}\mathbf{f}\mathbf{e}\mathbf{c}\mathbf{t}}={\mathbf{V}\mathbf{A}\mathbf{R}}_{1997-{2014}_{\mathbf{C}\mathbf{L}\mathbf{I}\mathbf{M}}}-{\mathbf{V}\mathbf{A}\mathbf{R}}_{1980-{1997}_{\mathbf{C}\mathbf{L}\mathbf{I}\mathbf{M}}}$$

$$\mathbf{G}\mathbf{R}\mathbf{A}\mathbf{Z}\mathbf{E}={\mathbf{O}\mathbf{V}\mathbf{E}\mathbf{R}\mathbf{A}\mathbf{L}\mathbf{L}}_{\mathbf{e}\mathbf{f}\mathbf{f}\mathbf{e}\mathbf{c}\mathbf{t}}-{\mathbf{N}\mathbf{o}\mathbf{g}\mathbf{r}\mathbf{a}\mathbf{z}\mathbf{i}\mathbf{n}\mathbf{g}}_{\mathbf{e}\mathbf{f}\mathbf{f}\mathbf{e}\mathbf{c}\mathbf{t}}$$

$$\mathbf{I}\mathbf{n}\mathbf{t}\mathbf{e}\mathbf{r}\mathbf{a}\mathbf{c}\mathbf{t}\mathbf{i}\mathbf{v}\mathbf{e}={\mathbf{O}\mathbf{v}\mathbf{e}\mathbf{r}\mathbf{a}\mathbf{l}\mathbf{l}}_{\mathbf{e}\mathbf{f}\mathbf{f}\mathbf{e}\mathbf{c}\mathbf{t}}-{\mathbf{C}\mathbf{L}\mathbf{I}\mathbf{M}}_{\mathbf{e}\mathbf{f}\mathbf{f}\mathbf{e}\mathbf{c}\mathbf{t}}-{\mathbf{C}\mathbf{O}}_{2\mathbf{e}\mathbf{f}\mathbf{f}\mathbf{e}\mathbf{c}\mathbf{t}}-{\mathbf{G}\mathbf{R}\mathbf{A}\mathbf{Z}\mathbf{E}}_{\mathbf{e}\mathbf{f}\mathbf{f}\mathbf{e}\mathbf{c}\mathbf{t}}$$

where VAR refers to TOTC, VEGC, SOC, and NPP, respectively, $\mathbf{I}\mathbf{n}\mathbf{t}\mathbf{e}\mathbf{r}\mathbf{a}\mathbf{c}\mathbf{t}\mathbf{i}\mathbf{v}\mathbf{e}$ means a dynamic interaction between climate, CO_2_ level, and grazing intensity changes. The years in the subscript indicate the time period, and the letter indicates the specific situation.

## Results

### AEM validation

In order to verify whether the AEM with the coupled grazing module more faithfully reflected grassland C dynamics, we compared its performance with the simulation provided by the initial AEM. Figure 3 indicates that NPP calculated by the parameterized AEM with grazing realistically matched the observed NPP for grazed grassland (*R*^2^ = 0.81, *p* < 0.05). The non-grazing data mainly came from the fence data collected from traditional pastures, which are protected from animal husbandry and grazing as much as possible. Although there was a lack of observed non-grazing NPP data, the simulation by using AEM with grazing yielded NPP for non-grazing grasslands that matched well the NPP in fenced sites (*R*^2^ = 0.46, *p* < 0.05). Compared with the original AEM, the AEM coupled with the grazing module captured 60% and 84% more change characteristics of the measured VEGC and SOC across sampling plots, respectively. The model tended to underestimate VEGC and slightly overestimated SOC. The discrepancy between the simulations and observations could be due to defects in the model structure, insufficient calibration of eco-physiological parameters, or uncertainties in the input data.

### Spatiotemporal changes of C dynamics

#### Temporal variation

**Fig. 4:**
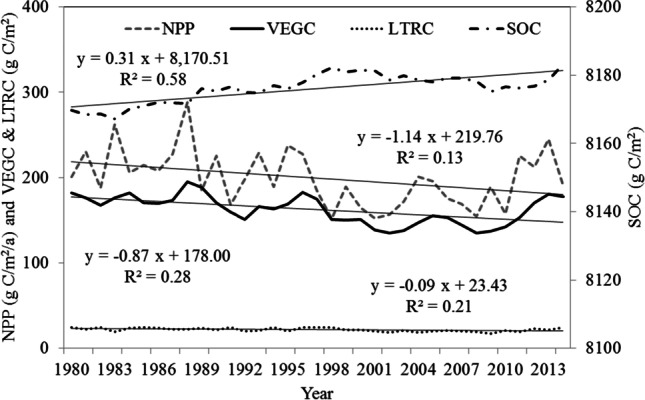
C pools and NPP changes in grassland ecosystem in Central Asia from 1980 to 2014

**Fig. 5:**
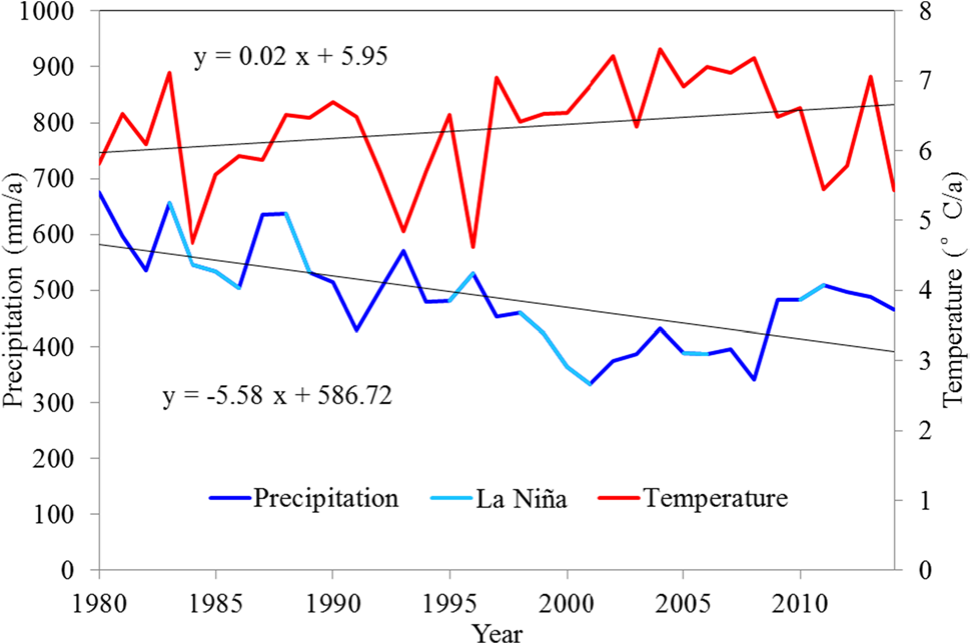
Temporal variations of annual precipitation and yearly mean temperature. La Niña events were recorded by Golden Gate Weather Services (http://ggweather.com/enso/oni.htm)

C dynamics in the investigated grasslands experienced significant temporal variations (Fig. 4). In the past 35 years, changes in NPP had an almost identical tendency to changes in VEGC (correlation coefficient *R* = 0.71). The NPP had a downward trend of − 1.14 g C/m^2^/a, accompanied by drastic interannual variability. The annual NPP variation remained steady between 1980 and 1996, but NPP was more variable from 1998 to 2008, with the minima recorded in 2001, 2006, and 2008, when major La Niña phenomena occurred (Fig. 5). Moreover, VEGC stocks declined from 182.22 to 177.82 g C/m^2^, with very low values between 1998 to 2008, when the precipitation was significantly lower, only accounting for 80.87% of the average rainfall over the studied period, and when the temperature was higher than the mean value of 0.62 °C. SOC stocks increased slightly from 8169.78 to 8,183.25 g C/m^2^. The fluctuation of SOC is primarily due to the dynamic interaction between litterfall carbon (LTRC) input and soil respiration consumption. The suppression of soil respiration triggered by the decline of soil moisture was larger than the effect of warmer temperatures.

**Fig. 6:**
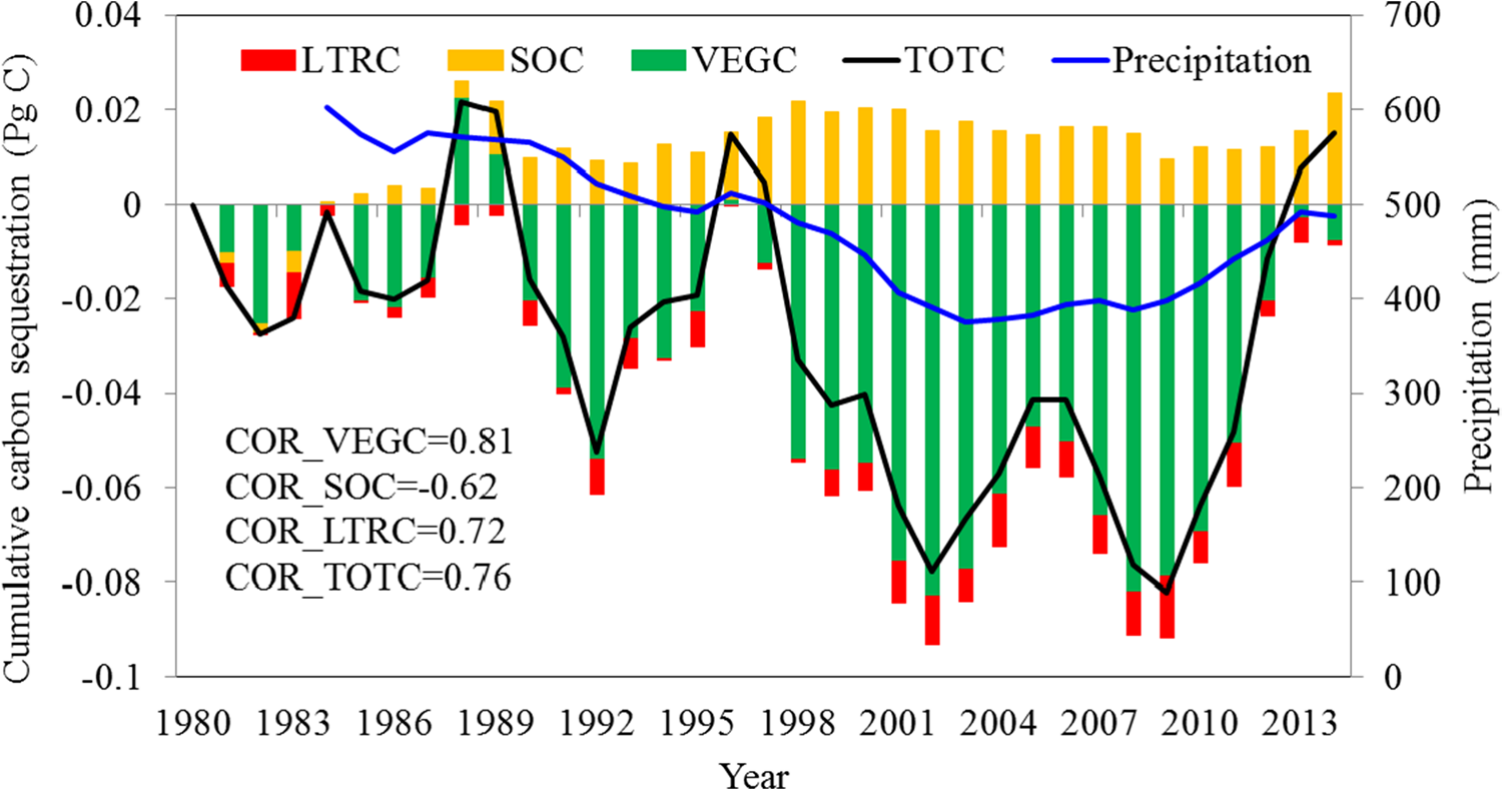
Cumulative C sequestration in different C pools relative to the levels recorded in 1980 in CA from 1980 to 2014. Precipitation is a 5-year moving average value. “Cor” indicates the correlation between precipitation and C pools

As shown in Fig. 6, the temporal pattern of the cumulative C sequestration, especially for TOTC and VEGC, closely followed the grassland annual precipitation fluctuations. In the past 35 years, the climate in CA showed a “warm-dry” trend, with an annual temperature increase by 0.02 °C/a and a decrease in precipitation − 5.58 mm/a (Fig. 5). There were significant positive correlations (*p* < 0.01) between the 5-year moving mean precipitation fluctuation and cumulative C sequestration in TOTC (*R* = 0.76), VEGC (*R* = 0.81), and LTRC (*R* = 0.72), respectively. A negative correlation between precipitation and SOC was also found (*R* =  − 0.62, *p* < 0.01). Temporal TOTC sequestration changes generally followed VEGC variations. In addition, although the SOC stocks were higher than those of 1980 during most years of the studied period, and the LTRC stocks were lower than those of 1980, their variation was small, and their contributions to grassland C dynamics was not obvious.

#### Spatial variation

**Fig. 7:**
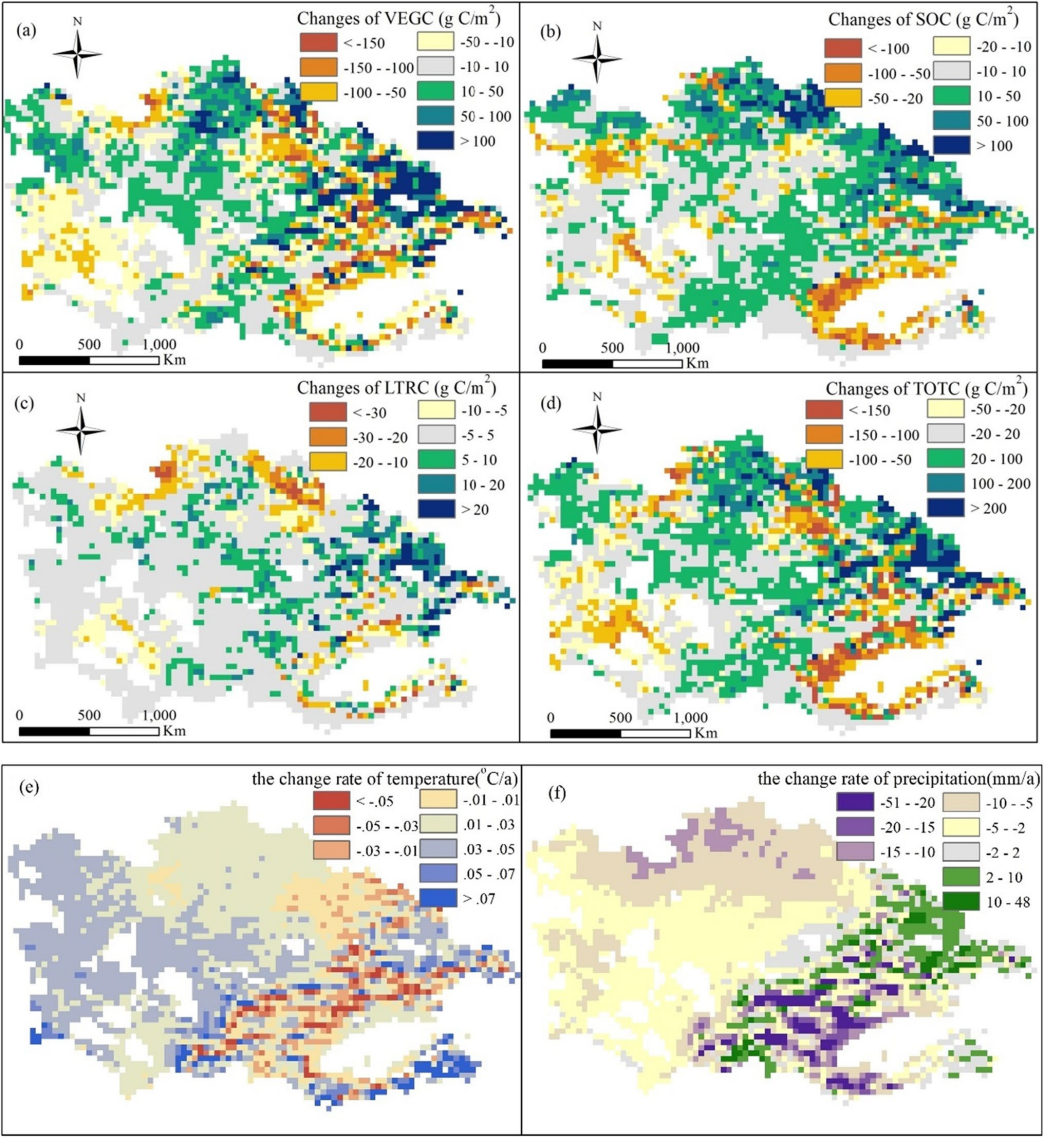
Spatial patterns of the change rate of different C pools (g C/m^2^), precipitation (mm/a), and temperature (°C/a). White space represents water or migrating dunes

Figure 7 shows the spatial pattern change from 1980 to 2014 of different C pools simulated with temporally varying environmental factors. Mountainous areas such as Tianshan and Altay Mountains showed strong carbon sink trends, especially in the middle and lower mountain belts. However, Southern Xinjiang, the Turgay Plateau of northern Kazakhstan, and western CA acted as a C source, where VEGC, SOC, and LTRC had different decreasing trends. Further analyses showed that in Southern Xinjiang, TOTC decreased by more than 100 g C/m^2^, likely owing to prolonged drought (Fig. 7) and associated grassland degradation and SOC loss (which decreased by more than 50 g C/m^2^). However, in regions with relatively abundant precipitation, e.g., the northern slope of the Tianshan Mountains, and with a slower temperature increase, e.g., grasslands in northern Kazakhstan, except for the Turgay Plateau areas, TOTC showed an increasing trend. In general, the precipitation variations strongly contributed to the spatial changes in the different C stocks.

### Relative contribution of the individual and interactive effects of different factors

**Fig. 8:**
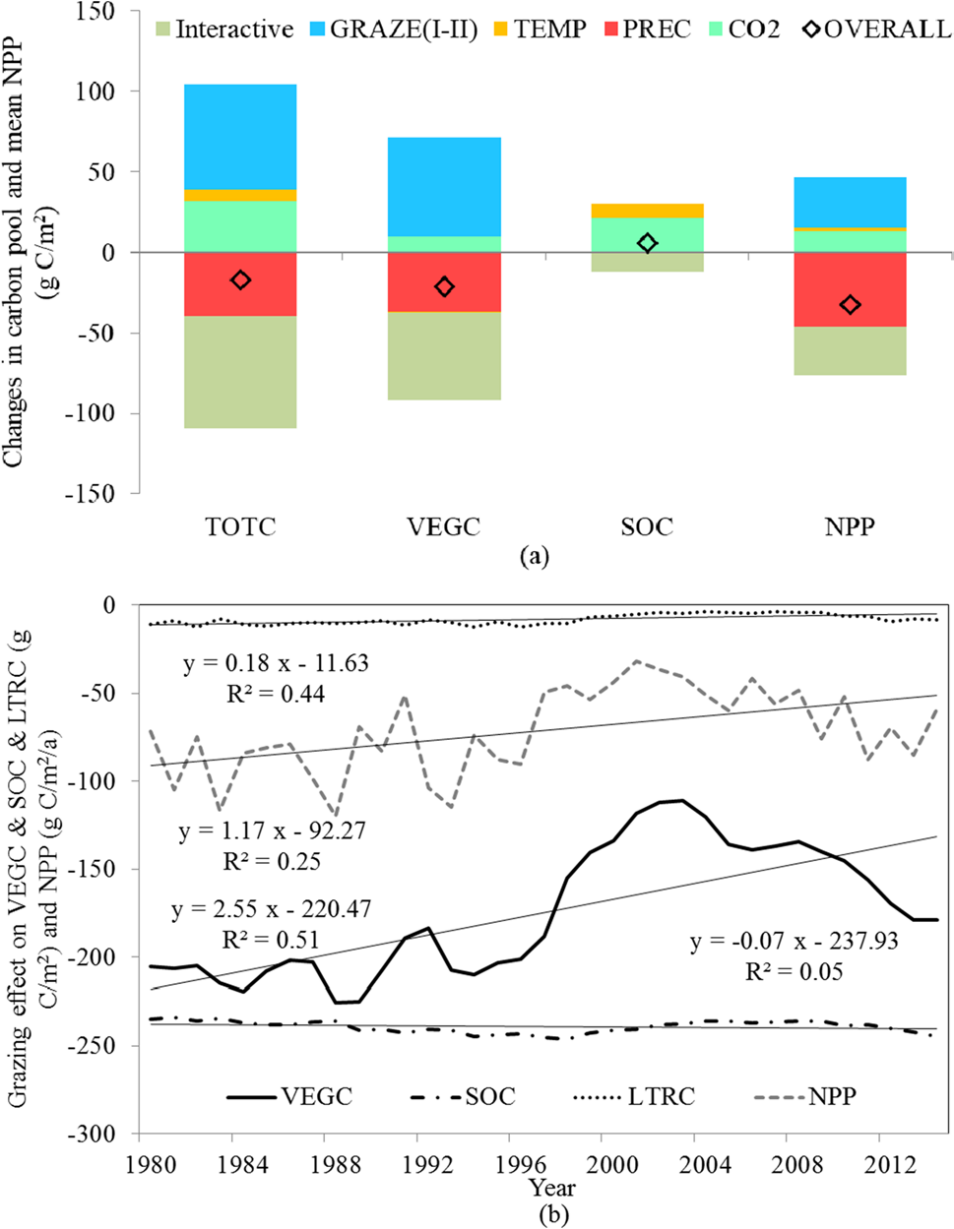
Factorial analysis: **a** individual and interactive effects of environmental factors on different C pools and NPP. **b** Interannual variation of grazing effect on VEGC, SOC, LTRC, and NPP from 1980 to 2014

Factor analysis was used to calculate the individual and interactive effects of various factors on TOTC, VEGC, SOC, and NPP (Fig. 8a). Affected by the combined effect of all environmental factors, TOTC decreased by 17.54 g C/m^2^, which was a slight reduction of 0.2%. Similarly, NPP and VEGC decreased by 32.52 g C/m^2^/a and 21.41 g C/m^2^, respectively, whereas SOC increased by 5.83 g C/m^2^. PREC showed that the negative effect of precipitation alone reduced TOTC in the grasslands by 39.95 g C/m^2^ (a 0.5% reduction), which was approximately sixfold and 1.25-fold higher than the positive effect of temperature change alone (TEMP) and CO_2_ enrichment (CO_2_), respectively. The positive effect of CO_2_ enrichment on SOC (21.15 g C/m^2^) was significantly larger than on VEGC (9.74 g C/m^2^) or NPP (12.79 g C/m^2^/a). Temperature changes had a complex influence on the dynamics of C pools. The slight positive effect of temperature variations on TOTC can generally be attributed to SOC variation. The grazing effect was calculated by comparing the OVERALL and no grazing scenarios. Grazing alone lowered TOTC by 422.19 g C/m^2^, VEGC by 174.60 g C/m^2^, SOC by 239.27 g C/m^2^, and NPP by 71.16 g C/m^2^/a from 1980 to 2014 (Fig. 8b). The results show that grazing declined the capacity for grassland C sequestration in CA. At the same time, we found that a decrease in grazing intensity had a positive effect on TOTC and NPP (Fig. 8a), and decreased grazing intensity, partly caused by the dissolution of the Soviet Union, led to the restoration of CA grasslands. The interactive effect of climate, CO_2_, and grazing intensity change decreased TOTC, VEGC, SOC, and NPP by 69.75 g C/m^2^, 53.82 g C/m^2^, 12.26 g C/m^2^, and 30.33 g C/m^2^/a, respectively.

### Spatial pattern of the grazing effect

**Fig. 9:**
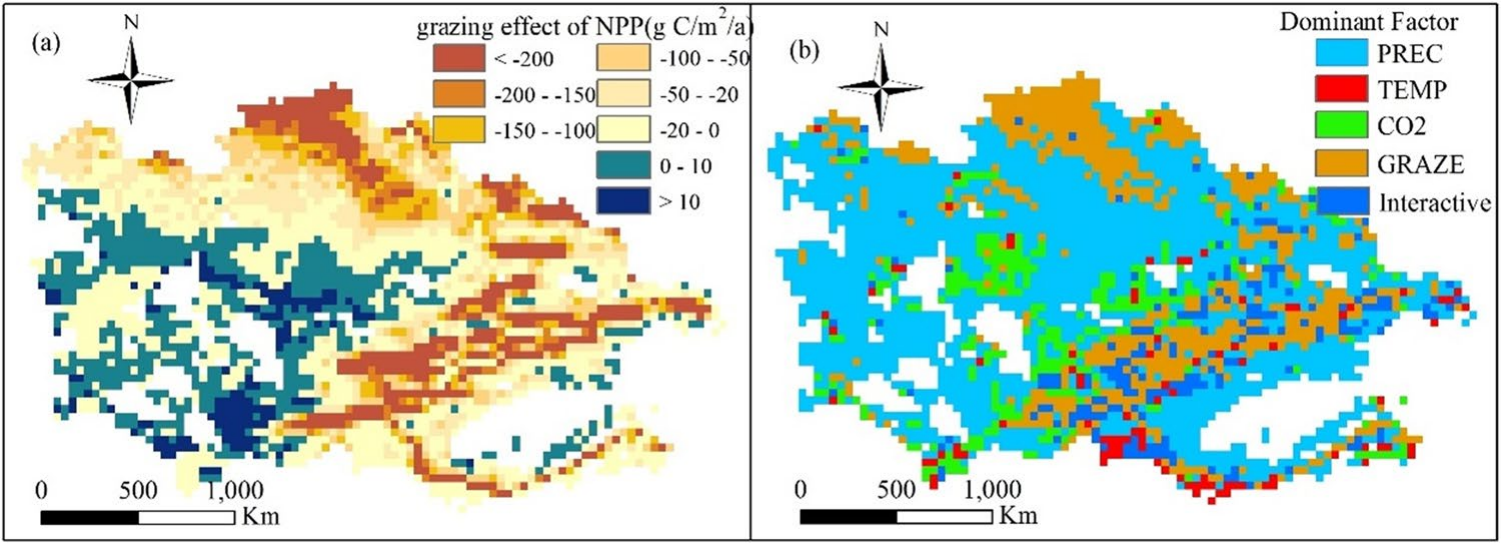
**a** Spatial pattern of the effect of grazing on NPP and **b** dominant factors that affected NPP in CA grasslands. A factor was considered as dominant if its impact on NPP was stronger than that of any other factor. PREC, TEMP, CO_2_, GRAZE, and Interactive indicate precipitation, temperature, CO_2_ level, grazing intensity change, and the interaction effect of climate, CO_2_ level, and grazing intensity changes, respectively

Figure 9 shows the spatial pattern of NPP comparative data under grazing and no grazing as well as dominant factors affecting NPP in the investigated grasslands. Previous studies defined overcompensation as the observation of greater NPP after grazing compared to that under the no-grazing condition, whereas undercompensation is the opposite phenomenon (Han et al. 2014). We found that overcompensation occurred in 25.71% of the studied area, mainly in the western part, where the annual average precipitation was 227.65 mm, annual average temperature was 12.65 °C, and average grazing intensity was 0.56 head/ha (Fig. 9a). We also observed that undercompensation occurred in most parts of the investigated grasslands, including the Tianshan Mountains and northern Kazakhstan grasslands. Although the grazing intensity with undercompensation (0.54 head/ha) was comparable to that with overcompensation (0.56 head/ha), the climate in areas with undercompensation significantly differed (annual precipitation of 518.44 mm and annual mean temperature of 5.49 °C) from that of the areas with overcompensation. These results indicated that compensatory growth was affected by the precipitation and botanic characteristics of the growing season. Different areas had different grazing carrying capacities under distinct environmental and climatic conditions.

To further identify the control factors that dominated the NPP changes in each grid of the grasslands, we developed a spatial map by comparing the impacts on NPP under different environmental factors (Fig. 9b). We found that precipitation was the most important climate factor. Approximately in 59% of the studied area, NPP was predominantly influenced by precipitation, primarily in southern Xinjiang and desert regions. The temperature effect dominated only in 3% of the studied area, including the alpine areas of the Tianshan and Kunlun Mountains. In turn, the CO_2_ effect dominated in 10% of the area, mainly in the northwest windward slope of the Tianshan, with abundant hydrothermal conditions. The grazing-dominated area was mostly located in pastures suitable for grazing in Northern Kazakhstan and the Tianshan Mountains, accounting for approximately 21% of the studied area. NPP in the remaining 7% of the region, distributed in the Tianshan Mountains, was mainly affected by the factor interaction effects.

## Discussion

### Implications of changes in CA grassland C dynamics under the influence of multiple factors

Our simulated grassland C dynamics were in good agreement with those of previous field observation and model simulation studies performed for CA areas (Table 3). The grasslands in CA acted as a weak TOTC source of 17.54 g C/m^2^, which was mainly caused by a decrease in VEGC of 21.41 g C/m^2^ and a slight increase of SOC of 5.83 g C/m^2^ during the past 35 years. C was mainly lost in southern Xinjiang and the Turgay Plateau in northern CA. This conclusion is supported by previous studies that indicated an increased rate of plant degradation in southern Xinjiang (Han et al. 2016) and Turgay Plateau (Zhang and Ren 2017). In addition, our modeling analysis indicated that NPP decreased by 15.0% in the investigated grasslands and was mainly affected by precipitation (Figs. 8a and 9b). In particular, the persistent drought (possibly related to the La Niña phenomenon) from the mid-1990s to the early 2000s caused a serious C loss in desert and grassland areas (de Beurs et al. 2009). The result is consistent with previous reports that indicated precipitation-dominated NPP changes in CA (Zhang and Ren 2017), which were not sensitive to temperature (Gang et al. 2015). Our study further demonstrated that the precipitation effect dominated in 59% of the studied region, whereas the temperature effect dominated only in 3% of CA territory, primarily in high latitudes and alpine mountains. It is predicted that warming rate in CA will be higher than the average value in the northern hemisphere in future, but precipitation patterns will show spatial heterogeneity (Lioubimtseva and Cole 2006; Huang et al. 2014). The rapid warming of grasslands in CA may not have a significant direct impact on NPP, whereas the indirect impact of the enhanced potential evapotranspiration and water stress on NPP may be considerable (Zhu et al. 2019). In addition, North Kazakhstan is the region with the fastest increase in precipitation in CA (3–9 mm/10a) (Huang et al. 2014), so that vegetation degradation caused by the continued drought in the Turgay plateau (Western Kazakhstan) may be alleviated in the future. Although there will be an increasing precipitation trend in Xinjiang in the future (by 20–35%), the rate of warming in Xinjiang is the highest in CA (5–6 °C) (compared with a temperature increase of 2–3 °C in the Turgay Plateau) (Mannig et al. 2013). Water stress caused by higher temperature may offset or even suppress the effect of increasing precipitation, which makes it difficult to alleviate vegetation degradation caused by drought in southern Xinjiang.

**Table 3:** Comparison of C pools (g C/m^2^) and productivity (g C/m^2^/a) between previous studies and this study

Study area	Methods	Result	Sources
Kazakhstan grassland	Modified LUE model	NPP: 131 to 205	(Propastin and Kappas 2009; Propastin et al. 2012)
Central Asia grassland	Arid ecosystem model	NPP: 171 ± 36	(Zhang and Ren 2017)
Dry steppe in Central Asia	Field observation	NPP: 126 to 326	(Perschina and Yakovlewa 1960; Makarowa 1971; Gristchenco 1972)
Central Asia grassland	Biome-BGC model	NPP: 158.14 to 194.69; SOC: 5736.86 VEGC: 61.72	(Han et al. 2016)
Central part of Eurasia	BEPS model and Shiyomi Grazing model	NPP: 79.5	(Chen et al. 2017b)
Central Asia grassland	Arid ecosystem model	VEGC: 400 ± 130; SOC: 6840 ± 4840	(Li et al. 2015)
	Field observation	VEGC: 500 ± 280; SOC: 5520 ± 3590	
Central Asia grassland	AEM_grazing model	VEGC: 162.27; SOC: 8176.13; NPP: 199.28	This study

### Effect of grazing on C dynamics

Because of the heterogeneous climate pattern and limited ecological data, quantitative assessments of grassland ecosystem responses to climate change and grazing disturbance in CA are scarce and difficult. In this study, we isolated and identified complex individual and interaction effects of a combination of environmental factors. For instance, we found a negative effect of grazing on NPP in the Tianshan Mountains and Northern Kazakhstan grasslands but a positive effect in the relatively dry western region of CA (Fig. 9). Although the two areas experienced similar grazing intensities (0.54 head/ha and 0.56 head/ha), the climate conditions were very different. We found that grazing under drought environmental stress stimulated grassland ecosystems to assimilate CO_2_ and decreased the capacity of ecosystems to assimilate CO_2_ during humid period and intermittent wet events. This finding is also supported by Long et al. (2019). Indeed, different durations of grazing periods, hydrothermal conditions, grassland types, and soil nutrients led to different responses of grassland ecosystems to grazing (Trlica and Rittenhouse 1993). For example, in Mongolia, Sim-CYCLE simulation results showed that aboveground biomass and NPP in grasslands decreased with increasing grazing intensity (Wu et al. 2008). However, in Inner Mongolia, before reaching the maximal C stock values (grazing intensity = 2.67 sheep/hm^2^), grassland NPP tended to increase with increasing grazing intensity, whereas precipitation attenuated or aggravated this changing trend (Wang 1998). In the central plain of the USA, which has a temperate continental climate (average annual rainfall of 446 mm), the aboveground grassland NPP was only affected by rainfall conditions but not by grazing intensity (Biondini et al. 1998). In the alpine Qinghai-Tibet Plateau (average annual rainfall of 600 mm), grazing promoted NPP in alpine meadows and reduced the negative effects of global warming on meadow NPP (Klein et al. 2007). We also found that adverse grazing effects could be compensated for by CO_2_ enrichment and improved climatic conditions. Adjusting grazing density according to future climate conditions is an inevitable requirement for the rational allocation of resources and sustainable ecosystem development.

Furthermore, multi-factor analysis helped to evaluate and measure the interaction effects of climate, CO_2_, and grazing factors. The interaction had a negative impact on NPP and TOTC, dominating mainly in the Tianshan Mountains, with strong climate variability and grazing changes. Several previous experiments not only confirmed our results that grazing can reduce the capacity of ecosystems to assimilate CO_2_ during wet years (Long et al. 2019) and that dryland plant TOTC tends to be insensitive to CO_2_ levels under long-term drought (Zhang and Ren 2017), but also supported the notion that the positive effect of CO_2_ enrichment comprised approximately 80% of the negative effect of precipitation variations. Although the compensation effect of CO_2_ can offset some negative effects of grazing, precipitation changes, and interaction effects on grassland C pools as the CO_2_ enrichment effect gradually decreases (Wang et al. 2020), the negative effects caused by climate change and grazing will increase significantly.

### Model uncertainty

The ecosystem model conceptualizes and abstracts complex biogeochemical processes by using relatively simple mathematical formulas or physical equations to describe various geological processes, which inevitably leads to uncertainties in the simulation results (Warszawski et al. 2013). As for the other factors influencing the C cycle, land use and land cover changes have been overlooked, although their role has been quite important in CA, especially after the collapse of the Soviet Union (de Beurs and Henebry 2004; Lioubimtseva and Henebry 2012). For example, large amounts of farmland in the northern part of Kazakhstan were abandoned and partially converted to grassland. According to statistical data, from 1991 to 2009, the farmland area in CA decreased by 22.03% (Li et al. 2015). Remote sensing observations indicated that farmland abandonment and grazing intensity change have led to an increase in vegetation greenness in CA (Wright et al. 2012). The AEM overlooks processes such as SOC depletion from reclamation (Sommer and de Pauw 2011) or the induced browning process by anthropogenic decisions (de Beurs et al. 2009; Wright et al. 2012). SOC depletion by water erosion, especially by wind erosion (Lal 2007), removed more than 40 g SOC m^−2^ in northern Xinjiang in the 1990s, and 5–15 g SOC m^−2^ a^−1^ in the Taklimakan Desert in southern Xinjiang (Yan et al. 2005). In addition, the AEM does not consider the effects of wind erosion and changes in land use on C dynamics.

Grazing trampling, excrement feedback, and defoliation play an important role in grassland ecosystems, but the AEM does not consider the impact of animal trampling. For example, animal trampling persisted throughout the grazing period. Due to the cumulative effect, stumped grass may reach up to 23% of the total grassland (Teng 2010). In addition, the impact of livestock trampling on grasslands may increase soil compaction (Weigel et al. 1990) and lower the capacity of soils to hold water (Kobayashi et al. 1997), affecting soil humus and N accumulation (Severson and Debano 1991). Therefore, establishing the trampling index in the model and quantifying trampling intensity in the future will have important practical significance for grassland monitoring and improvement of degraded grasslands in CA.

## Conclusions

The AEM with a grazing module was used in this study to investigate C dynamics under the influence of various climate factors and grazing management in CA. The results show that C dynamics in CA are mainly affected by precipitation. The area in which C dynamics was mainly controlled by temperature comprised only ~ 3% of CA. Grazing had a negative effect on NPP and C stocks, and overcompensation occurred in 25.71% of CA area, mainly in the western part of the investigated grasslands. The adverse impact of grazing was compensated by CO_2_ enrichment. The complex interaction effects of climate factors, CO_2_ level, and grazing had negatively influenced NPP. Overall, the NPP in CA grassland had a declining tendency of − 1.14 g C/m^2^/a. The temporal curve of the cumulative C sequestration closely related to the annual precipitation change. Our simulations showed that southern Xinjiang and the Turgay Plateau of Kazakhstan are ecologically fragile areas due to serious degradation of NPP. In the context of future climate change, exploring grazing mechanisms and setting grazing safety thresholds are key measures to ensure sustainable development of grasslands in CA.

## Data Availability

All data generated or analyzed during this study are included in this article.
